# Digital Transformation of Healthcare in Lebanon: A Strategic Response to Health System Fragility

**DOI:** 10.34172/ijhpm.9482

**Published:** 2026-05-03

**Authors:** Mohamad Ali Hijazi

**Affiliations:** Department of Pharmaceutical Sciences, Faculty of Pharmacy, Beirut Arab University, Beirut, Lebanon.

## Introduction

 The Lebanese health system has been weakened by a cascading and prolonged economic and financial crises because of combination of economic collapse, political instability, repeated crises, and the residual impacts of protracted conflict.^1^ These factors have led to depreciation of the national currency, severe supply-chain disruptions, interruptions in drug availability, and large-scale emigration of physicians, pharmacists, nurses, and allied health staff.^2^ The situation is further exacerbated by the immense financial pressures on hospitals, compounded by inadequate government support and the absence of universal health insurance. Hospitals and primary care centers face escalating operational costs and reduced capacity to maintain equipment and critical services.^3^ The already fragile state of Lebanon’s healthcare infrastructure, weakened by decades of repeated wars, internal strife, and political instability, serves only to deepen the crisis. The Lebanese Order of Pharmacists has reported the closure of more than 700 pharmacies during the past years.^4^ Adding to this dire scenario are alarmingly high rates of burnout among healthcare professionals which have exceeded 40% in some healthcare professions.^4^

 This aim of this article is not to provide a policy-oriented Viewpoint developed by the academic and professional engagement of the author with health system and profession in Lebanon during ongoing crisis. It synthesizes illustrative evidence from both stable and fragile settings, using focused searches of suitable literature and policy reports to inform a context-sensitive analysis. For that, digital health transformation in Lebanon should not be viewed as a comprehensive system reform or technological “rescue.” Rather, it should be recognized as a pragmatic, phased, and adaptive strategy aimed at mitigating service disruption, preserving workforce capacity, and stabilizing access in underserved regions.

## Definition of Digital Health

 According to the World Health Organization (WHO), digital health represents a broader umbrella encompassing electronic health records, mobile health applications, and health information systems, and other technology-enabled health services.^5^ Telehealth refers specifically to the remote provision of healthcare services and clinical support through telecommunications technologies, including telemedicine consultations, tele-pharmacy services, and remote patient monitoring.^6^ In this manuscript, the term “digital health” is used broadly, while specific modalities (telehealth, telemedicine, tele pharmacy) are distinguished where relevant.

## Evolution of Digital Health Across Health System Contexts

 Contrary to the assumption that it is a recent innovation, digital health date back to the 1960s, when NASA pioneered telemedicine for monitoring astronauts’ health.^7^ It was later adopted in rural areas of the United States and expanded in Canada during the 1990s, particularly in remote provinces. However, the development of digital health has evolved differently across health system contexts. Countries like United Kingdom and Australia have embedded digital and remote pharmacy services within nationally coordinated reimbursement and care pathways.^8^ In the Arab world, the adoption of digital health has varied according to political stability, infrastructure capacity, and governmental support. The United Arab Emirates the Kingdom of Saudi Arabia have integrated telehealth into formal health strategies and insurance coverage frameworks.^9^

 The COVID-19 pandemic catalyzed rapid global adoption of telehealth services to sustain care under crisis conditions.^10^ However, these implementations occurred within relatively stable governance, financing, and infrastructure environments that differ strongly from Lebanon’s health system fragility. In contrast, in fragile or crisis-affected settings, telehealth has often emerged as a compensatory mechanism to alleviate service disruption and workforce shortages, where significant implementation barriers were evident. In countries like Iraq, Yemen, and Syria, telehealth initiatives have faced interruptions due to electricity instability, weak regulatory oversight, digital illiteracy, and fragmented financing structures.^11^

## The Status of Lebanon: Challenges and Opportunities

###  Challenges Facing the Healthcare System

 Multiple reports from Lebanon documented severe shortage of many essential medicines (including chronic medications) in community pharmacies during the crisis.^12^ Recent studies suggest that approximately 40% of Lebanese patients were unable to access necessary healthcare services in 2023 due to financial constraints or difficulty reaching healthcare providers.^13^ Furthermore, over two million individuals from vulnerable populations, including refugees, are dependent on already overburdened primary healthcare centers.^14^ These challenges are exacerbating with the ongoing emigration of skilled healthcare professionals, creating a severe brain drain within the system.

###  System Readiness for Digital Health

 Despite these formidable challenges, the conditions in Lebanon are particularly conducive to the implementation of digital solutions. Reports reveal that 75% of Lebanese physicians utilized telemedicine during the COVID-19 pandemic.^15^ Prior to the pandemic, over 54% of psychotherapists were already employing telehealth technologies.^15^ Coupled with the widespread adoption of smartphones and internet access, encompassing over 80% of the population, and the public’s demonstrated trust in digital applications during the COVID-19 crisis (as evidenced by the successful adoption of the Ministry of Public Health’s [MOPH’s] “Ma’an” application and COVID-19 tracking tools), Lebanon appears at good position to embrace digital health solutions.^16^

## The Transformative Potential of Digital Health in Lebanon

###  Enhancing Efficiency and Reducing Costs 

 It is worthy to mention that reported benefits of telemonitoring programs from high-income may not be directly transferable to Lebanon without supportive institutional conditions.^8^ In Canada, for example, a remote pharmacy service saved approximately US$ 100 000 annually in staffing costs within a rural community without compromising the quality of patient care. Moreover, data indicate that patient adherence to prescribed medications for chronic conditions (like diabetes and hypertension) improved by 20%-25% through remote monitoring and follow-up interventions.^17^

###  Promoting Equity in Access to Healthcare

 Digital health systems enable patients in remote, underserved areas to directly access healthcare services, addressing critical needs in Lebanon’s border regions and mountainous terrains. These systems also offer healthcare accessibility outside of traditional working hours, accommodating evenings, weekends, and public holidays. Digital health solutions are particularly valuable in situations where physical access is hindered due to road closures caused by snow, political unrest, fuel shortages, or armed conflict, all too common occurrences in Lebanon. Furthermore, digital health services reduce transportation costs for low-income individuals and contribute to alleviating congestion and long waiting times in private clinics and healthcare centers.^6^

###  Improving Health Outcomes and Reducing Morbidity and Mortality

 Countries that have effectively integrated digital health services into their healthcare systems report a significant decrease in mortality and complications associated with chronic illnesses. For example, remote patient monitoring has resulted in an absolute reduction of 0.5-1.0 percentage points in hemoglobin A1c levels in diabetic patients. In the field of mental health, tele-cognitive behavioral therapy and psychological monitoring have been proven to reduce relapse rates and the need for hospitalization. Telehealth services also contribute to the long-term management of blood pressure and lipid levels, reducing the risk of strokes and cardiovascular events.^18^

###  Alleviating the Strain on the Healthcare System

 Many Lebanese patients report difficulties finding available beds in hospitals, leading to delays in receiving necessary care and increased risk of complications. This situation is exacerbated by the ongoing treatment of individuals injured in past conflicts and the displacement of communities from destroyed towns and villages. Digital health services can help alleviate pressure on hospitals, emergency rooms, and healthcare centers, allowing them to prioritize complex cases and maintain high-quality care.^19^

###  Improving Resilience of Healthcare Workers 

 Importantly, digital health initiatives can decrease burnout among healthcare professionals, as evidenced by several reports. It allows healthcare providers to cover underserved areas without physical relocation. It also enables them to serve a larger patient population especially specialty physicians and those managing rare conditions.^8^

 While international evidence highlights potential benefits, improved chronic disease management, and cost reductions, the magnitude of these benefits in Lebanon will be questionable with its infrastructural fragility, governance fragmentation, and economic volatility. In this case, digital health may primarily serve as a stabilizing instrument to preserve continuity of care rather than a driver of rapid cost savings or large-scale efficiency gains.

## Challenges and Barriers to Implementation

###  Regulatory and Legal Gaps

 Currently, there is no comprehensive legal framework formally governing telehealth and tele-pharmacy practice, including professional liability, licensure recognition, reimbursement eligibility, and informed consent procedures. Concerns regarding data protection and the absence of a coordinated health information governance structure further limit applicability.^6,8,20^

###  Infrastructure and Operational Constraints 

 Persistent electricity instability, disturbed internet connectivity, and fragmented digital platforms will equally challenge the operational feasibility. Unlike developed settings where telehealth operates within stable utilities and integrated electronic records, Lebanon’s infrastructural fragility, currency volatility, and macroeconomic instability may increase short-term implementation costs, making financial projections uncertain.

###  Governance Fragmentation 

 The healthcare system is characterized by fragmented authority, pluralistic financing arrangements, and overlapping public and private actors with differing incentives. Action like increased transparency in prescribing patterns, referral pathways, and reimbursement mechanisms are examples of essential power dynamics. Technological innovation cannot compensate alone without regulatory consistency and accountability.

###  Digital Literacy and Workforce Readiness 

 Disparities in digital literacy particularly among older adults and rural populations may further limit equitable access, underscoring the need for structured capacity building among both patients and providers.

## Recommendations for Digital Health Implementation in Lebanon

 Given Lebanon’s abovementioned challenges, digital health expansion should follow a phased and adaptive strategy rather than immediate decision led solely by the MOPH. Implementation may follow a phased technological progression, beginning with low-bandwidth teleconsultations and tele-pharmacy services, followed by remote monitoring initiatives, and eventually integration with efficient electronic health platforms. Altohugh the Ministry remains a critical regulatory actor, early implementation may require collaborative efforts with universities, professional orders, non-governmental organizations, private insurers, and diaspora networks.

###  Phase I: Pilot-Tested and Structurally Integrated Actions

####  a. Enable Provisional Regulatory Recognition

 The MOPH could issue interim regulatory guidance formally recognizing teleconsultation and tele-pharmacy under defined pilot conditions. This would clarify professional accountability, informed consent procedures, and basic data protection standards while avoiding delays associated with full parliamentary reform.

####  b. Launch Pilot Programs in Underserved Regions

 Pilot telehealth clinics could be introduced in structurally underserved areas such as Hermel, Rashaya, Akkar, and conflict-affected areas in Southern Lebanon. These pilots should prioritize chronic disease follow-up, mental health services, and medication management areas where continuity gaps are most pronounced.

####  c. Tele-Pharmacy Test within the Primary Healthcare Centers

 A controlled pilot in 5-10 governmental healthcare centers could assess feasibility of remote pharmacist consultations and supervised digital dispensing, particularly in rural areas or regions suffering from pharmacist shortages.

####  d. Implementation in Selected University Hospitals 

 Lebanese university hospitals represent a best choice for early implementation. It can provide training, technical oversight, and quality assurance. Moreover, universities can mitigate governance fragmentation by offering structured supervision and data evaluation capacity.

###  Phase II: Hybrid Financing and Capacity Development

####  e. Initial Hybrid Reimbursement

 Because of financial constraints within national insurance schemes, pilot reimbursement mechanisms could involve private insurers, funded programs, or co-payment models before gradual integration into National Social Security Fund structures.

####  f. Structured Capacity Building

 Professional health orders, universities, and non-governmental organization should collaborate to train healthcare workers in digital triage, teleconsultation protocols, and referral criteria. Capacity building should precede scale-up to ensure service efficiency, safety and quality.

####  g. Diaspora Engagement Platforms

 Digital health platforms will engage the Lebanese diaspora clinicians, particularly for specialist consultations not widely available in rural regions. Clear credentialing and accountability mechanisms would be required.

###  Phase III: Monitoring, Evaluation, and Gradual Institutionalization

####  h. Develop Context-Sensitive Monitoring Frameworks

 Initial evaluation of pilot programs should prioritize feasibility metrics including service uptake, chronic care continuity, patient satisfaction, and infrastructure reliability rather than focusing long term clinical outcomes such as hospital readmissions rates. As institutional stability improves, more outcome-based key performance indicators may be incorporated. Over time, successful pilot initiatives could inform more comprehensive legislative reform and integration into national health strategy.

 The perspective reflects the author’s academic and professional experience. Future efforts should utilize established implementation science frameworks, such as Reach Effectiveness Adoption Implementation Maintenance and Consolidated Framework for Implementation Research to ensure systematic evaluation and long-term sustainability.

## Limitations

 This Viewpoint does not constitute a systematic review or formal economic evaluation. The gathered data is illustrative and selected to contextualize policy considerations rather than to provide exhaustive synthesis. Transferability of outcomes from stable health systems to fragile contexts remains uncertain. In addition, the analysis reflects the authors’ professional and academic perspectives within the Lebanese health sector and may not reflect all stakeholder viewpoints. Future empirical research and stakeholder consultations are necessary to validate feasibility assumptions.

## Conclusion

 Digital health in Lebanon should be framed as a pragmatic instrument for stabilizing a fragile healthcare system rather than as a transformative cure. In the context of institutional fragmentation, economic collapse, and workforce migration, its realistic contribution lies in preserving continuity of care and mitigating service deterioration rather than delivering rapid efficiency gains. Implementation will require phased deployment, regulatory clarity, and alignment with existing institutional capacities. While digital innovation cannot substitute for broader governance reform, carefully sequenced and context-sensitive interventions may incrementally strengthen resilience and support the gradual restoration of functional stability within the health sector.

## Disclosure of artificial intelligence (AI) use

 AI was used only for editing grammar and spelling mistakes of the text.

## Ethical issues

 Not applicable.

## Conflicts of interest

 Author declares that he has no conflicts of interest. The author is affiliated with academic institutions and has no financial ties to telehealth companies or governmental digital health programs.
